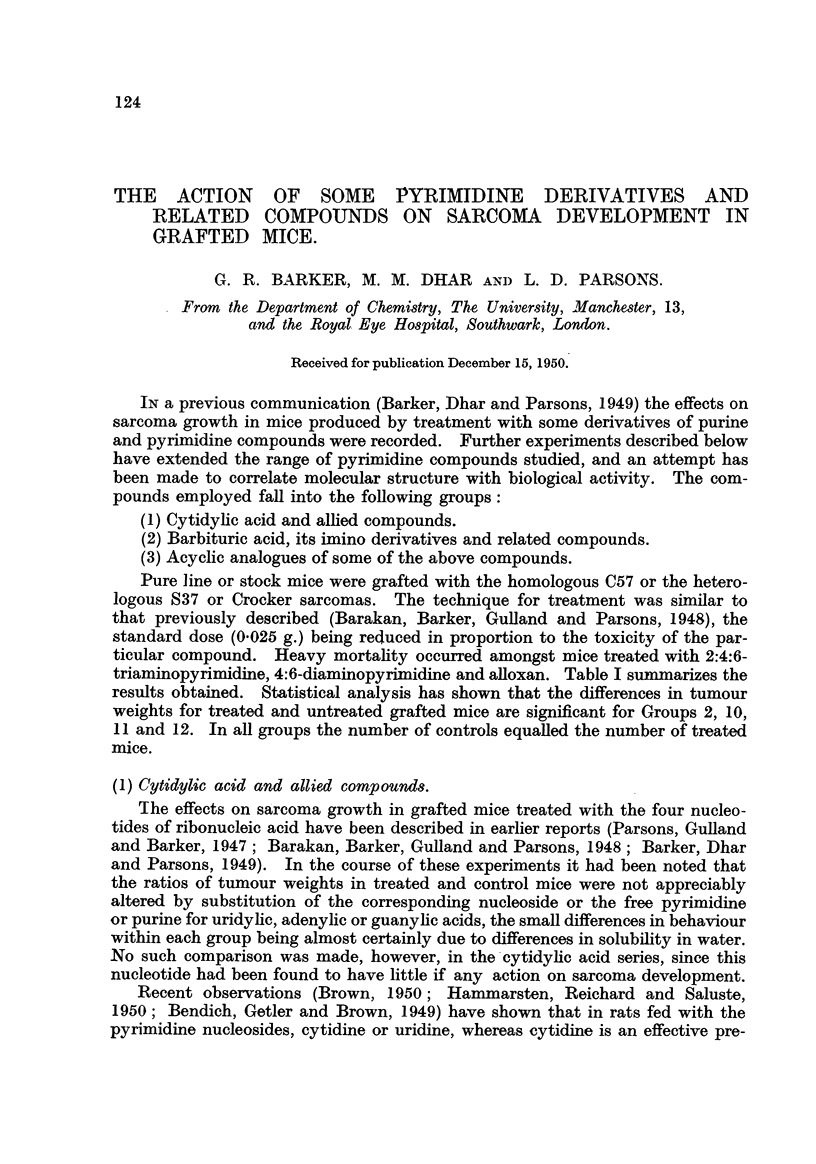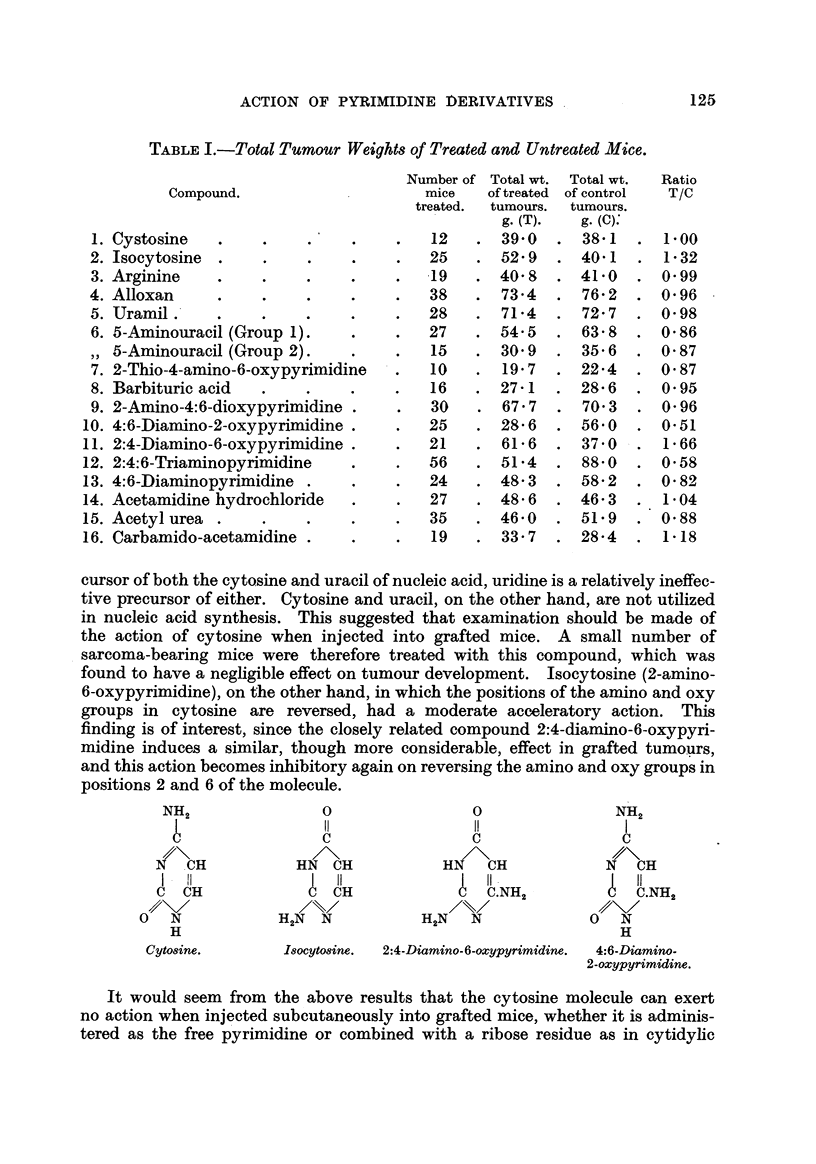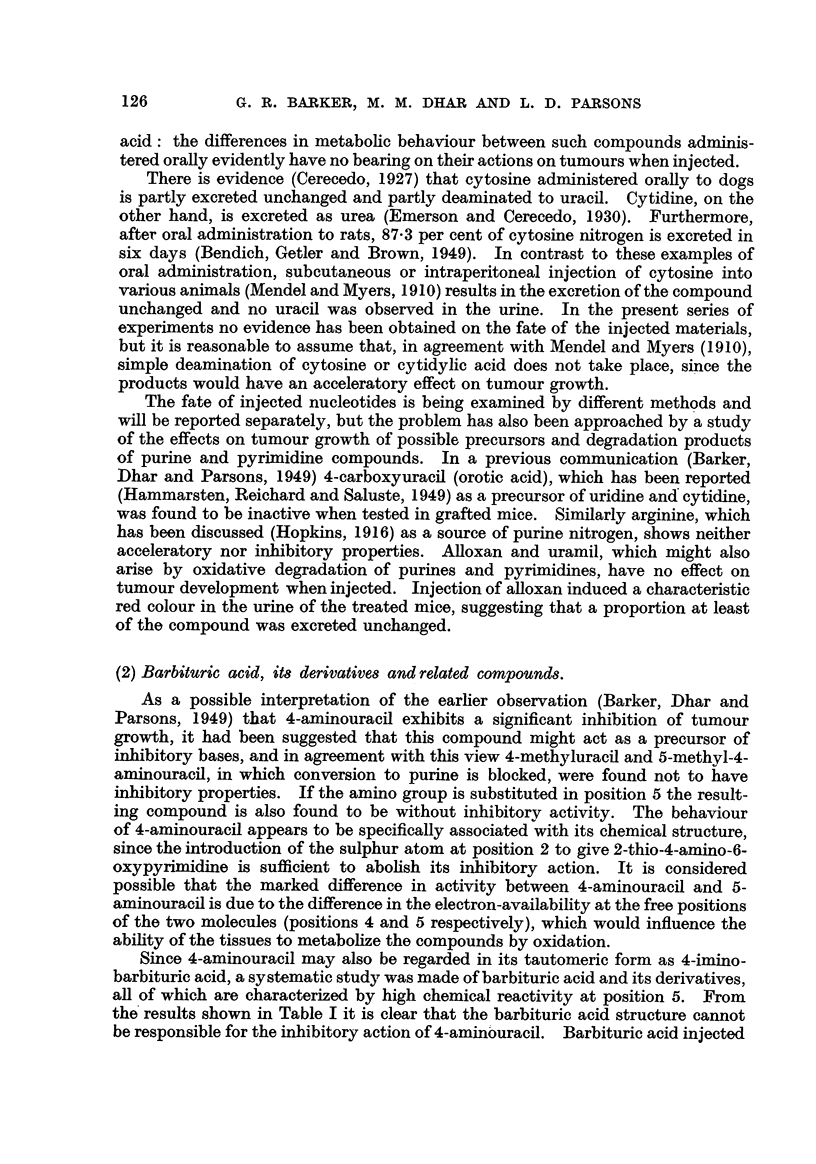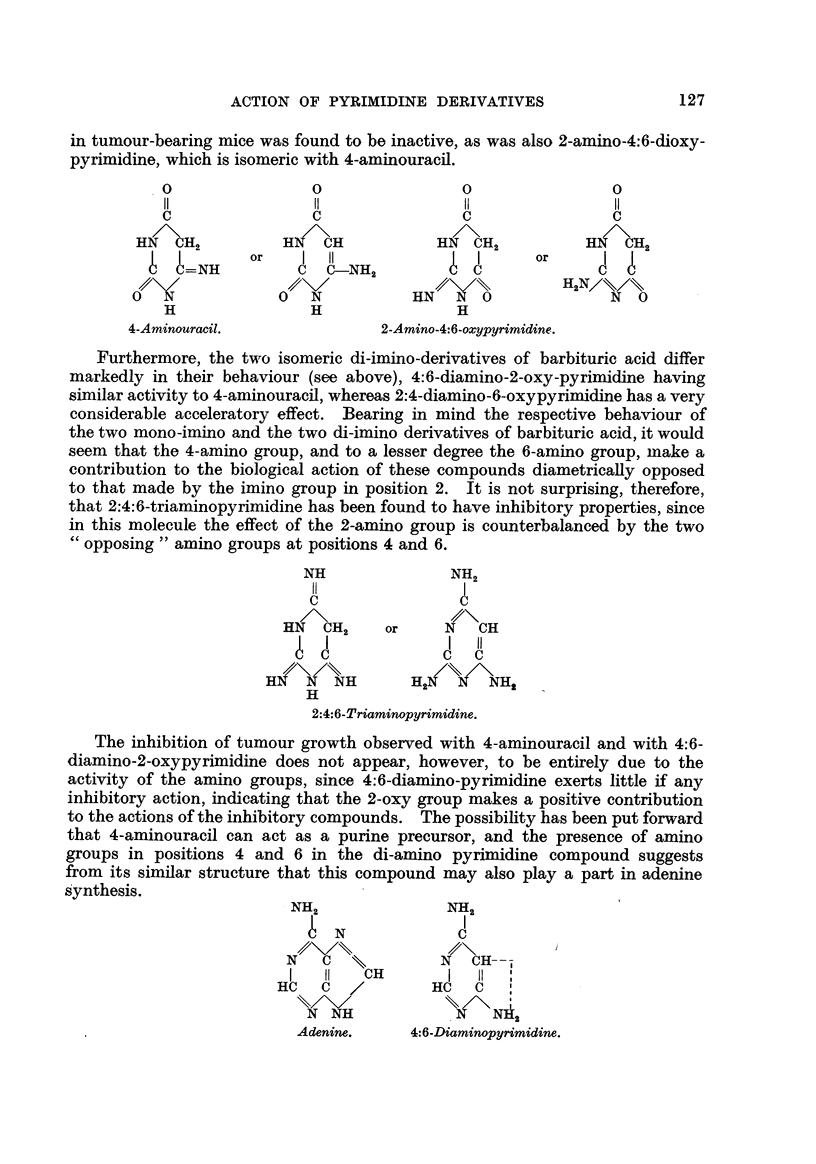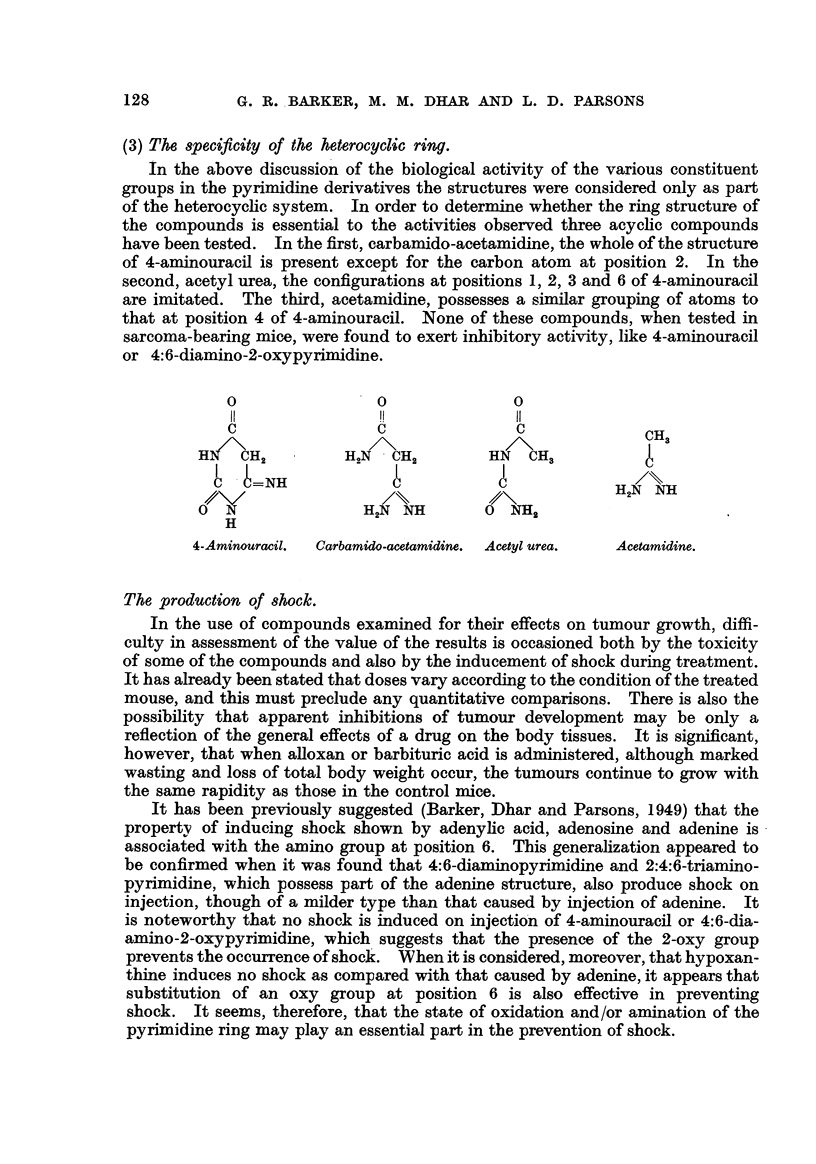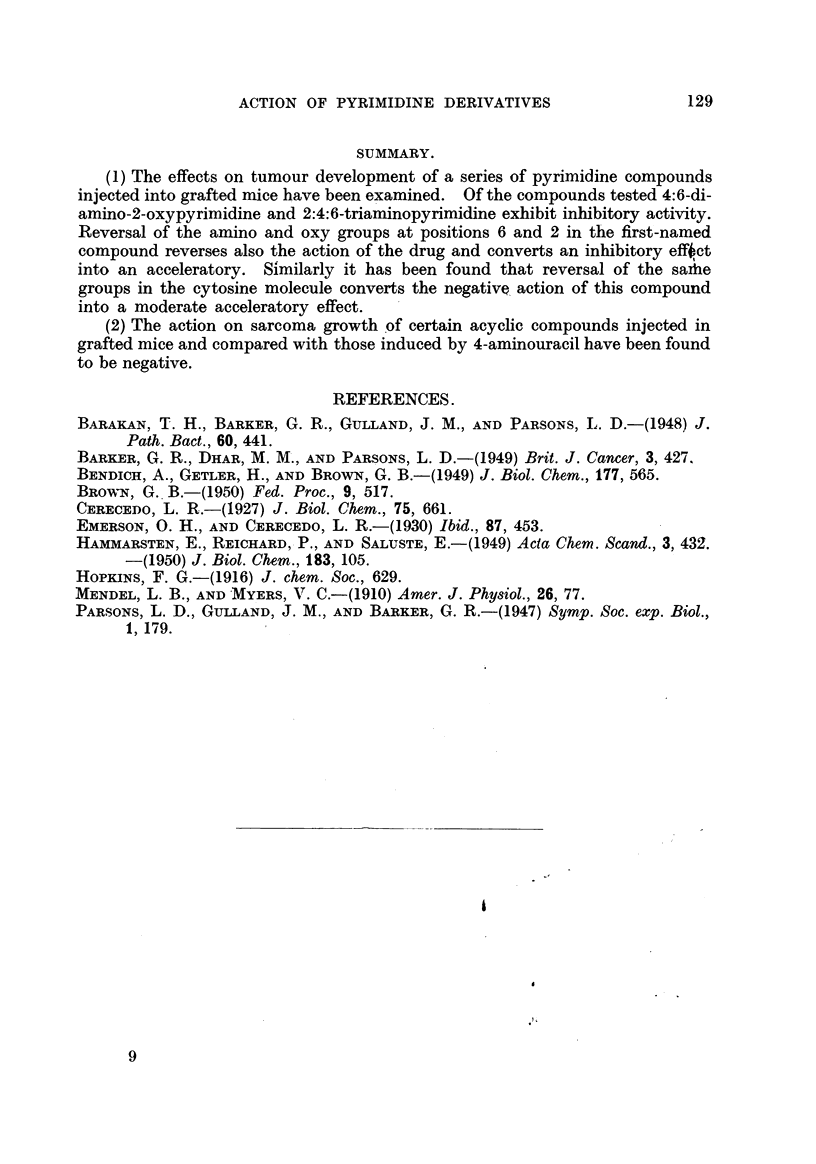# The Action of Some Pyrimidine Derivatives and Related Compounds on Sarcoma Development in Grafted Mice

**DOI:** 10.1038/bjc.1951.12

**Published:** 1951-03

**Authors:** G. R. Barker, M. M. Dhar, L. D. Parsons


### THE ACTION OF SOME PYRIMIDINE DERIVATIVES AND RELATED COMPOUNDS ON SARCOMA DEVELOPMENT IN GRAFTED MICE.

G. R. BARKER, M. M. DHAR AND L. D. PARSONS.

*From the Department of Chemistry, The University, Manchester, 13, and the Royal Eye Hospital, Southwark, London.*

Received for publication December 15, 1950.

In a previous communication (Barker, Dhar and Parsons, 1949) the effects on sarcoma growth in mice produced by treatment with some derivatives of purine and pyrimidine compounds were recorded. Further experiments described below have extended the range of pyrimidine compounds studied, and an attempt has been made to correlate molecular structure with biological activity. The compounds employed fall into the following groups :

(1) Cytidylic acid and allied compounds.
(2) Barbituric acid, its imino derivatives and related compounds.
(3) Acyclic analogues of some of the above compounds.

Pure line or stock mice were grafted with the homologous C57 or the heterologous S37 or Crocker sarcomas. The technique for treatment was similar to that previously described (Barakan, Barker, Gulland and Parsons, 1948), the standard dose (0·025 g.) being reduced in proportion to the toxicity of the particular compound. Heavy mortality occurred amongst mice treated with 2:4:6-triaminopyrimidine, 4:6-diaminopyrimidine and alloxan. Table I summarizes the results obtained. Statistical analysis has shown that the differences in tumour weights for treated and untreated grafted mice are significant for Groups 2, 10, 11 and 12. In all groups the number of controls equalled the number of treated mice.

(1) *Cytidylic acid and allied compounds.*

The effects on sarcoma growth in grafted mice treated with the four nucleotides of ribonucleic acid have been described in earlier reports (Parsons, Gulland and Barker, 1947 ; Barakan, Barker, Gulland and Parsons, 1948 ; Barker, Dhar and Parsons, 1949). In the course of these experiments it had been noted that the ratios of tumour weights in treated and control mice were not appreciably altered by substitution of the corresponding nucleoside or the free pyrimidine or purine for uridylic, adenylic or guanylic acids, the small differences in behaviour within each group being almost certainly due to differences in solubility in water. No such comparison was made, however, in the cytidylic acid series, since this nucleotide had been found to have little if any action on sarcoma development.

Recent observations (Brown, 1950 ; Hammarsten, Reichard and Saluste, 1950 ; Bendich, Getler and Brown, 1949) have shown that in rats fed with the pyrimidine nucleosides, cytidine or uridine, whereas cytidine is an effective pre-

TABLE I.—*Total Tumour Weights of Treated and Untreated Mice.*

| Compound. | Number of mice treated. | Total wt. of treated tumours. g. (T). | Total wt. of control tumours. g. (C). | Ratio T/C |
|---|---|---|---|---|
| 1. Cystosine | 12 | 39·0 | 38·1 | 1·00 |
| 2. Isocytosine | 25 | 52·9 | 40·1 | 1·32 |
| 3. Arginine | 19 | 40·8 | 41·0 | 0·99 |
| 4. Alloxan | 38 | 73·4 | 76·2 | 0·96 |
| 5. Uramil | 28 | 71·4 | 72·7 | 0·98 |
| 6. 5-Aminouracil (Group 1) | 27 | 54·5 | 63·8 | 0·86 |
| ,, 5-Aminouracil (Group 2) | 15 | 30·9 | 35·6 | 0·87 |
| 7. 2-Thio-4-amino-6-oxypyrimidine | 10 | 19·7 | 22·4 | 0·87 |
| 8. Barbituric acid | 16 | 27·1 | 28·6 | 0·95 |
| 9. 2-Amino-4:6-dioxypyrimidine | 30 | 67·7 | 70·3 | 0·96 |
| 10. 4:6-Diamino-2-oxypyrimidine | 25 | 28·6 | 56·0 | 0·51 |
| 11. 2:4-Diamino-6-oxypyrimidine | 21 | 61·6 | 37·0 | 1·66 |
| 12. 2:4:6-Triaminopyrimidine | 56 | 51·4 | 88·0 | 0·58 |
| 13. 4:6-Diaminopyrimidine | 24 | 48·3 | 58·2 | 0·82 |
| 14. Acetamidine hydrochloride | 27 | 48·6 | 46·3 | 1·04 |
| 15. Acetyl urea | 35 | 46·0 | 51·9 | 0·88 |
| 16. Carbamido-acetamidine | 19 | 33·7 | 28·4 | 1·18 |

cursor of both the cytosine and uracil of nucleic acid, uridine is a relatively ineffective precursor of either. Cytosine and uracil, on the other hand, are not utilized in nucleic acid synthesis. This suggested that examination should be made of the action of cytosine when injected into grafted mice. A small number of sarcoma-bearing mice were therefore treated with this compound, which was found to have a negligible effect on tumour development. Isocytosine (2-amino-6-oxypyrimidine), on the other hand, in which the positions of the amino and oxy groups in cytosine are reversed, had a moderate acceleratory action. This finding is of interest, since the closely related compound 2:4-diamino-6-oxypyrimidine induces a similar, though more considerable, effect in grafted tumours, and this action becomes inhibitory again on reversing the amino and oxy groups in positions 2 and 6 of the molecule.

*Cytosine.*

*Isocytosine.*

2:4-*Diamino*-6-*oxypyrimidine.*

4:6-*Diamino*-2-*oxypyrimidine.*

It would seem from the above results that the cytosine molecule can exert no action when injected subcutaneously into grafted mice, whether it is administered as the free pyrimidine or combined with a ribose residue as in cytidylic acid: the differences in metabolic behaviour between such compounds administered orally evidently have no bearing on their actions on tumours when injected.

There is evidence (Cerecedo, 1927) that cytosine administered orally to dogs is partly excreted unchanged and partly deaminated to uracil. Cytidine, on the other hand, is excreted as urea (Emerson and Cerecedo, 1930). Furthermore, after oral administration to rats, 87·3 per cent of cytosine nitrogen is excreted in six days (Bendich, Getler and Brown, 1949). In contrast to these examples of oral administration, subcutaneous or intraperitoneal injection of cytosine into various animals (Mendel and Myers, 1910) results in the excretion of the compound unchanged and no uracil was observed in the urine. In the present series of experiments no evidence has been obtained on the fate of the injected materials, but it is reasonable to assume that, in agreement with Mendel and Myers (1910), simple deamination of cytosine or cytidylic acid does not take place, since the products would have an acceleratory effect on tumour growth.

The fate of injected nucleotides is being examined by different methods and will be reported separately, but the problem has also been approached by a study of the effects on tumour growth of possible precursors and degradation products of purine and pyrimidine compounds. In a previous communication (Barker, Dhar and Parsons, 1949) 4-carboxyuracil (orotic acid), which has been reported (Hammarsten, Reichard and Saluste, 1949) as a precursor of uridine and cytidine, was found to be inactive when tested in grafted mice. Similarly arginine, which has been discussed (Hopkins, 1916) as a source of purine nitrogen, shows neither acceleratory nor inhibitory properties. Alloxan and uramil, which might also arise by oxidative degradation of purines and pyrimidines, have no effect on tumour development when injected. Injection of alloxan induced a characteristic red colour in the urine of the treated mice, suggesting that a proportion at least of the compound was excreted unchanged.

(2) *Barbituric acid, its derivatives and related compounds.*

As a possible interpretation of the earlier observation (Barker, Dhar and Parsons, 1949) that 4-aminouracil exhibits a significant inhibition of tumour growth, it had been suggested that this compound might act as a precursor of inhibitory bases, and in agreement with this view 4-methyluracil and 5-methyl-4-aminouracil, in which conversion to purine is blocked, were found not to have inhibitory properties. If the amino group is substituted in position 5 the resulting compound is also found to be without inhibitory activity. The behaviour of 4-aminouracil appears to be specifically associated with its chemical structure, since the introduction of the sulphur atom at position 2 to give 2-thio-4-amino-6-oxypyrimidine is sufficient to abolish its inhibitory action. It is considered possible that the marked difference in activity between 4-aminouracil and 5-aminouracil is due to the difference in the electron-availability at the free positions of the two molecules (positions 4 and 5 respectively), which would influence the ability of the tissues to metabolize the compounds by oxidation.

Since 4-aminouracil may also be regarded in its tautomeric form as 4-imino-barbituric acid, a systematic study was made of barbituric acid and its derivatives, all of which are characterized by high chemical reactivity at position 5. From the results shown in Table I it is clear that the barbituric acid structure cannot be responsible for the inhibitory action of 4-aminouracil. Barbituric acid injected in tumour-bearing mice was found to be inactive, as was also 2-amino-4:6-dioxy-pyrimidine, which is isomeric with 4-aminouracil.

*4-Aminouracil.*

*2-Amino-4:6-oxypyrimidine.*

Furthermore, the two isomeric di-imino-derivatives of barbituric acid differ markedly in their behaviour (see above), 4:6-diamino-2-oxy-pyrimidine having similar activity to 4-aminouracil, whereas 2:4-diamino-6-oxypyrimidine has a very considerable acceleratory effect. Bearing in mind the respective behaviour of the two mono-imino and the two di-imino derivatives of barbituric acid, it would seem that the 4-amino group, and to a lesser degree the 6-amino group, make a contribution to the biological action of these compounds diametrically opposed to that made by the imino group in position 2. It is not surprising, therefore, that 2:4:6-triaminopyrimidine has been found to have inhibitory properties, since in this molecule the effect of the 2-amino group is counterbalanced by the two " opposing " amino groups at positions 4 and 6.

*2:4:6-Triaminopyrimidine.*

The inhibition of tumour growth observed with 4-aminouracil and with 4:6-diamino-2-oxypyrimidine does not appear, however, to be entirely due to the activity of the amino groups, since 4:6-diamino-pyrimidine exerts little if any inhibitory action, indicating that the 2-oxy group makes a positive contribution to the actions of the inhibitory compounds. The possibility has been put forward that 4-aminouracil can act as a purine precursor, and the presence of amino groups in positions 4 and 6 in the di-amino pyrimidine compound suggests from its similar structure that this compound may also play a part in adenine synthesis.

*Adenine.*

*4:6-Diaminopyrimidine.*

(3) *The specificity of the heterocyclic ring.*

In the above discussion of the biological activity of the various constituent groups in the pyrimidine derivatives the structures were considered only as part of the heterocyclic system. In order to determine whether the ring structure of the compounds is essential to the activities observed three acyclic compounds have been tested. In the first, carbamido-acetamidine, the whole of the structure of 4-aminouracil is present except for the carbon atom at position 2. In the second, acetyl urea, the configurations at positions 1, 2, 3 and 6 of 4-aminouracil are imitated. The third, acetamidine, possesses a similar grouping of atoms to that at position 4 of 4-aminouracil. None of these compounds, when tested in sarcoma-bearing mice, were found to exert inhibitory activity, like 4-aminouracil or 4:6-diamino-2-oxypyrimidine.

*4-Aminouracil.*

*Carbamido-acetamidine.*

*Acetyl urea.*

*Acetamidine.*

*The production of shock.*

In the use of compounds examined for their effects on tumour growth, difficulty in assessment of the value of the results is occasioned both by the toxicity of some of the compounds and also by the inducement of shock during treatment. It has already been stated that doses vary according to the condition of the treated mouse, and this must preclude any quantitative comparisons. There is also the possibility that apparent inhibitions of tumour development may be only a reflection of the general effects of a drug on the body tissues. It is significant, however, that when alloxan or barbituric acid is administered, although marked wasting and loss of total body weight occur, the tumours continue to grow with the same rapidity as those in the control mice.

It has been previously suggested (Barker, Dhar and Parsons, 1949) that the property of inducing shock shown by adenylic acid, adenosine and adenine is associated with the amino group at position 6. This generalization appeared to be confirmed when it was found that 4:6-diaminopyrimidine and 2:4:6-triaminopyrimidine, which possess part of the adenine structure, also produce shock on injection, though of a milder type than that caused by injection of adenine. It is noteworthy that no shock is induced on injection of 4-aminouracil or 4:6-diaamino-2-oxypyrimidine, which suggests that the presence of the 2-oxy group prevents the occurrence of shock. When it is considered, moreover, that hypoxanthine induces no shock as compared with that caused by adenine, it appears that substitution of an oxy group at position 6 is also effective in preventing shock. It seems, therefore, that the state of oxidation and/or amination of the pyrimidine ring may play an essential part in the prevention of shock.

SUMMARY.

(1) The effects on tumour development of a series of pyrimidine compounds injected into grafted mice have been examined. Of the compounds tested 4:6-diamino-2-oxypyrimidine and 2:4:6-triaminopyrimidine exhibit inhibitory activity. Reversal of the amino and oxy groups at positions 6 and 2 in the first-named compound reverses also the action of the drug and converts an inhibitory effect into an acceleratory. Similarly it has been found that reversal of the same groups in the cytosine molecule converts the negative action of this compound into a moderate acceleratory effect.

(2) The action on sarcoma growth of certain acyclic compounds injected in grafted mice and compared with those induced by 4-aminouracil have been found to be negative.

### REFERENCES.

Barakan, T. H., Barker, G. R., Gulland, J. M., and Parsons, L. D.—(1948) *J. Path. Bact.*, **60**, 441.

Barker, G. R., Dhar, M. M., and Parsons, L. D.—(1949) *Brit. J. Cancer*, **3**, 427.

Bendich, A., Getler, H., and Brown, G. B.—(1949) *J. Biol. Chem.*, **177**, 565.

Brown, G. B.—(1950) *Fed. Proc.*, **9**, 517.

Cerecedo, L. R.—(1927) *J. Biol. Chem.*, **75**, 661.

Emerson, O. H., and Cerecedo, L. R.—(1930) *Ibid.*, **87**, 453.

Hammarsten, E., Reichard, P., and Saluste, E.—(1949) *Acta Chem. Scand.*, **3**, 432. —(1950) *J. Biol. Chem.*, **183**, 105.

Hopkins, F. G.—(1916) *J. chem. Soc.*, 629.

Mendel, L. B., and Myers, V. C.—(1910) *Amer. J. Physiol.*, **26**, 77.

Parsons, L. D., Gulland, J. M., and Barker, G. R.—(1947) *Symp. Soc. exp. Biol.*, **1**, 179.